# Evolution of Highly Polymorphic T Cell Populations in Siblings with the Wiskott-Aldrich Syndrome

**DOI:** 10.1371/journal.pone.0003444

**Published:** 2008-10-20

**Authors:** Maxim I. Lutskiy, Jun Y. Park, Susanna K. Remold, Eileen Remold-O'Donnell

**Affiliations:** 1 Immune Disease Institute, Harvard Medical School, Boston, Massachusetts, United States of America; 2 Department of Pediatrics, Harvard Medical School, Boston, Massachusetts, United States of America; 3 Program on Disease Evolution, Department of Biology, University of Louisville, Louisville, Kentucky, United States of America; Columbia University, United States of America

## Abstract

Population level evolutionary processes can occur within a single organism when the germ line contains a mutation that confers a cost at the level of the cell. Here we describe how multiple compensatory mutations arose through a within-individual evolutionary process in two brothers with the immune deficiency Wiskott-Aldrich Syndrome (WAS). As a result, both brothers have T lymphocyte populations that are highly polymorphic at the locus of the germ line defect, and no single allele achieves fixation. WASP, the gene product affected in this disease, is specific to white blood cells where it is responsible for regulating actin cytoskeleton dynamics in a wide range of cellular responses. The brothers inherited a rare allele predicted to result in truncated WASP lacking the carboxy-terminal VCA domains, the region that directly catalyzes actin filament generation. Although the brothers' T cell populations are highly polymorphic, all share a corrective effect relative to the inherited allele in that they restore the VCA domain. This indicates massive selection against the truncated germ line allele. No single somatic allele becomes fixed in the circulating T cell population of either brother, indicating that a regulated step in maturation of the affected cell lineage is severely compromised by the germ line allele. Based on the finding of multiple somatic mutations, the known maturation pathway for T-lineage cells and the known defects of T cells and precursor thymocytes in mice with truncated WASP, we hypothesize that the presence of truncated WASP (WASPΔVCA) confers an extreme disadvantage in early developing thymocytes, above and beyond the known cost of absence of full-length WASP, and that the disadvantage likely occurs through dominant negative competition of WASPΔVCA with N-WASP, a protein that otherwise partially compensates for WASP absence in developing thymocytes.

## Introduction

The cells comprising a multicellular organism reproduce and compete in particular local environments, much as do free-living organisms. When genetic differences arise within a cell type, evolution can ensue within the host organism. The classic example of this phenomenon is cancer – a somatic mutant arises and increases in frequency relative to cells of the germ line genotype due to a selective advantage in the local environment. In cancer, selection at the level of the cell type differs from selection at the level of the host organism because the role of the affected cell type in the multicellular organism is at odds with maximizing the number of its progeny cells.

We present an instance involving a germ line allele causing disease at the level of the whole organism. In contrast to cancer causing mutations, in this situation the somatic mutations that increase in frequency due to selection at the level of the cell also confer benefit to the whole organism. This coincident benefit means that identifying the strength, timing and local environment in which the somatic mutation(s) confer advantage will contribute to a better understanding of how the affected cell type normally functions within the multicellular organism.

For genetic hematopoietic diseases, somatic mutations that are corrective at the level of the whole organism may have a special propensity to reach high frequency in the cell population. This is because a successful somatic mutant with higher fitness relative to hematopoietic cells carrying the inherited allele has long survival and a long-term influence on the organism's fate. In addition, hematopoietic cells function throughout the body, allowing compensatory somatic mutants to affect the organism systemically. However somatic mutations can only benefit the whole organism to the extent that *(i)* at the level of the cell type, selection on a newly arising somatic mutant is strongly positive and *(ii)* that selection against the germ line allele occurs early enough in the cell lineage that sufficient divisions remain for a mutation-corrected allele to increase in frequency in the cell population.

Here we describe a situation where the balance of these two factors is such that not one, but many, somatic mutations are detected. This occurred independently and in parallel in the T cell populations of two brothers who share the same germ line mutation of the gene *Wiskott Aldrich Syndrome protein (WASP*). We document these numerous compensatory somatic alleles and explore the timing, location and the nature of their selective advantage, thereby deducing that the gene product, also called WASP, affects T cell populations in at least two distinct contexts.

### Wiskott-Aldrich Syndrome (WAS) and WASP Function

Wiskott-Aldrich syndrome (WAS) (MIM #30100) is an X-linked combined primary immunodeficiency disease [1] causing bleeding due to thrombocytopenia and numerous immune defects (reviewed in [2]). Histocompatibility-matched hematopoietic stem cell transplantation is currently the only cure [3] for WAS. For patients for whom no histocompatible matched donor is available, life expectancy is still short, particularly for patients with WASP-negative mutations [4]. In part for this reason, WAS is among those conditions for which hematopoietic stem cell gene therapy is under intense study [5], [6].

Immune dysfunction in this disease varies from mild to life-threatening, depending to a large extent on whether the inherited mutation supports expression of some level of WASP in the patient's blood cells (milder disease) or leads to WASP absence (severe disease) [4]. In unusual cases, the impact of severe germ line *WASP* mutations is mitigated by somatic reversion or somatic second site mutation [7]–[13].

WASP couples extracellular stimulation of the cell to rapid intracellular remodeling of the actin-based cytoskeleton (reviewed in [14]). WASP is expressed only in white blood cells; its closely related homolog N-WASP is broadly expressed. Both proteins assume an autoinhibited (closed) conformation in resting cells [15]. WASP's function in cytoskeletal rearrangement is best understood in T lymphocytes. In these cells activation occurs when antigens bind to the T cell antigen receptor (T cell receptor; TCR). WASP is then recruited to the newly generated immunological synapse between the antigen presenting cell and the T cell [16]. At the immune synapse, activating mediators such as the lipid phosphoinositol 4,5-biphosphate (PIP2) and cdc42, a GTPase, bind to WASP, releasing autoinhibition and unmasking WASP's C-terminal VCA (verprolin homology, cofilin homology, acidic) domains. The VCA domains cooperate with a complex of actin related proteins (Arp2/3 complex) to generate new actin filaments [15], [17] that are required for altering cell polarity, proliferative response and further cell differentiation. Through this process, WASP is critical for optimal proliferative response of T lymphocytes and in a similar manner for B lymphocytes. WASP is also expressed in other white blood cells: monocytes, neutrophils, NK lymphocytes and platelets, where its actin remodeling activity contributes to migration, phagocytosis and other functions (reviewed in [14]).

WASP is also believed to contribute to maturation of progenitor T and B lymphoid cells. This inference rests on a cross-sectional study showing that WAS patients have decreased numbers of mature T and B lymphocytes in their blood relative to age-matched healthy normal individuals [18]. The deficit of total blood T and B cells primarily reflects the decrease of the initially produced antigen-inexperienced “naïve” T and B cells rather than antigen-experienced “memory” cells.

The brothers studied here inherited a rare *WASP* mutation (G1305 delete) predicted to result in a truncated protein that lacks the VCA domains (WASPΔVCA). Their mother is a confirmed carrier. As is typical for WAS carriers, her mature blood cells are normal in number and phenotype, reflecting selective survival and/or proliferation of progenitor cells with the active X-chromosome bearing normal *WASP* [19]. In the three other patients reported to carry this mutation (two of whom were maternal uncles of these brothers), the mutation caused severe WAS [20]. In contrast, the brothers reached adulthood with only moderate disease. Here we describe their somatic alleles and investigate their selective advantage in T-lymphopoiesis. Because during T-lymphopoiesis selection for survival and replication at the level of the thymocyte is concordant with these cells' normal contribution to the whole organism's phenotype, this selection sheds light on the normal role of WASP in T-lymphopoiesis.

## Results

### Identifying Cell Types Experiencing Selection for WASP Function

To resolve the discordancy of the brothers' severe germ line mutation and their moderate disease course, we subjected their blood cells to quantitative and qualitative analyses. This was done by staining blood samples with antibodies to cell surface “marker” proteins and analyzing the stained cells by flow cytometry. Table 1 shows the quantitation of their lymphocytes compared to age-matched normal healthy individuals. The results reflect the previously described pattern for this disease albeit at the more extreme end: the brothers share significant deficits of T cells (both CD4 and CD8) and B cells but have normal numbers of natural killer cells (NK cells) [18]. In particular, their memory CD4 and CD8 T cells were in the normal range, but the number of their naïve, *i.e.*, antigen-inexperienced, CD4 and CD8 cells were decreased by more than 75% relative to normal values. Notably, the patterns in the two brothers are almost identical.

**Table 1:** Lymphocyte Populations in Patient Blood.

Cells	Absolute cell count (×10^6^/ml)		
	Patient 1	Patient 2	Normal^a^ ^b^
Total lymphocytes	0.96	1.08	1.68 (1.48–2.00)*
T cells (CD3^+^ CD45^+^)	0.61	0.67	1.26 (1.04–1.52)*
B cells (CD19^+^ CD45^+^)	0.04	0.06	0.24 (0.18–0.26)**
NK cells (CD16^+^ CD56^+^ CD3^−^)	0.30	0.20	0.18 (0.13–0.37)^ns^
Naïve CD4 cells (CD45RA^+^)	0.09	0.10	0.40 (0.14–0.74)*
Memory CD4 cells (CD45RA^−^)	0.32	0.49	0.42 (0.32–0.48)^ns^
Naïve CD8 cells (CD45RA^+^CCR7^+^)	0.005	0.006	0.22 (0.18–0.34)*
Memory CD8 cells^c^	0.165	0.204	0.11 (0.09–0.21)^ns^

a Normal values (median;5^th^–95^th^ percentile) for 11 healthy adult males [18].

b Significance from one-tailed t-tests comparing cell counts of the normal healthy males to the brothers' counts: ^ns^ p>0.05; * 0.01<p<0.05; ** 0.001<p<0.01.

c Memory CD8 cells are the sum of CD8^+^CD45RA^+^CCR7^−^ and CD8^+^CD45RA^−^ cells.

**Figure 1:**
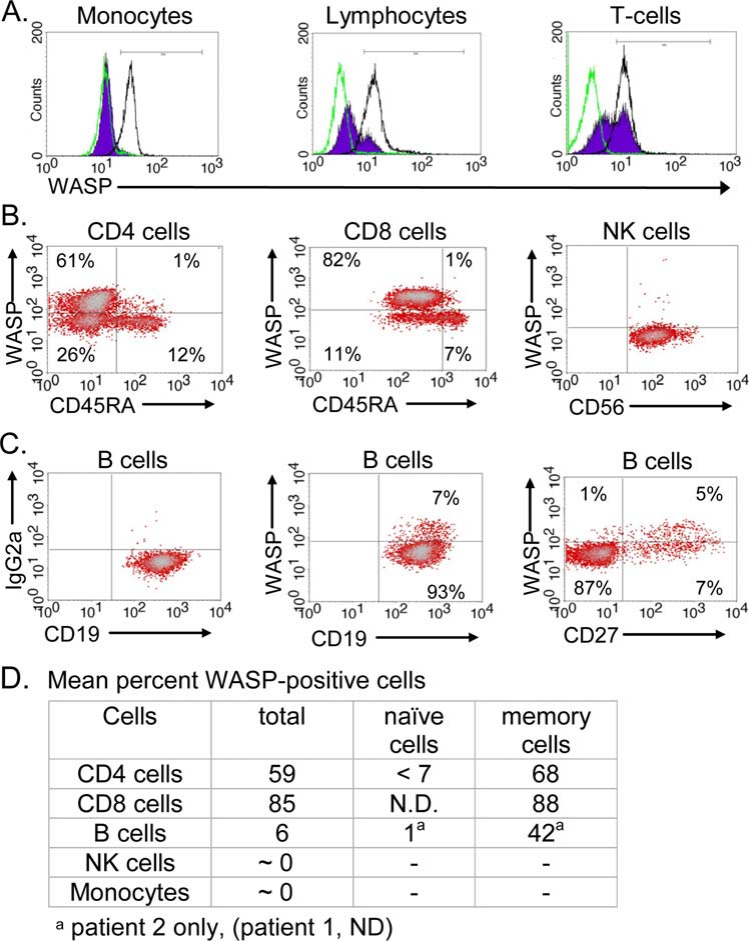
WASP in Patient Blood Cells Revealed by Flow Cytometry. PBMC of the younger brother (patient 2) were stained intracellularly with B9 monoclonal antibody to WASP and, where indicated, were stained with surface marker antibodies. (A) Monocytes and lymphocytes. Shown are patient monocytes (left), total lymphocytes (middle), and T lymphocytes (CD3^+^ cells) (right) indicated by filled histograms; cells of a normal individual are indicated by black outlines and isotype control by green outlines. (B) T and NK lymphocytes. CD4 (CD4^+^) and CD8 (CD8^bright^) cells, and NK cells (CD56^+^CD3^−^) stained for WASP and, where indicated, for CD45RA to distinguish T cell subsets. Numbers within quadrants indicate the percentage of cells. (C) B-lymphocytes. B cells (CD19^+^) were stained with isotype control antibody (left) or WASP antibody (middle, right) and for CD27 to distinguish B-cell subsets (right). (D) Shown are the percent WASP-positive cells in different populations averaged across the two brothers. ND, not determined.

Because cells of previously studied WAS patients with truncation mutations have revealed little or no WASP [4], [21], we used flow cytometry to determine whether any WASP is expressed in the brothers' cells. Peripheral blood mononuclear cells (PBMC) were permeabilized, stained intracellularly with fluorescent antibodies to WASP, and analyzed to detect presence *vs.* absence of the WASP protein at the level of single cells. Whereas monocytes of the brothers were WASP-negative, appearing unstained or dimly stained, their lymphocytes included a minor population of brightly staining WASP-positive cells (Figure 1A, left and middle). WASP-positive cells were more prevalent among T lymphocytes (CD3^+^ cells), comprising 56% for the younger brother (patient 2) (Figure 1A, right) and 58% for his sibling (patient 1, not shown). Of note, the intensity of fluorescent label, an approximate measure of WASP density, was comparable for the WASP-positive subset of patient T cells and normal T cells (Figure 1A, right), suggesting that patient cells that express WASP do so at normal or near normal levels. These T lymphocytes must either express more truncated WASP than other cells bearing the same genotype or they are WASP-positive because they bear a somatic allele.

WASP-positive cells were found in both CD4 and CD8 T-cells (Figure 1B, left and middle). Antibody to CD45RA, a stage-specific T cell antigen, was used to distinguish naïve and memory cells [22]. WASP-positive cells were frequent among memory CD4 cells (CD45RA^−^, upper left quadrant) (65% for patient 1; 70% for patient 2), but were infrequent (≤7%) among naive CD4 cells (CD45RA^+^, upper right quadrant). WASP-positive cells were also frequent among CD8 memory (CD45RA^−^) cells of both patients (89%, 88%) and infrequent in the small CD8^+^CD45RA^+^ population that includes the CD8 naïve cells. NK lymphocytes (NK cells) were uniformly WASP-negative (Figure 1B, right). These quantitative findings for the two brothers are compiled in Figure 1D.

A fraction of B lymphocytes (CD19^+^ lymphocytes) were also WASP-positive, amounting to 7% for patient 2 (Figure 1C, left and middle) and 4% for patient 1 (not shown). For patient 2, the B cells were further subdivided. WASP-positive cells were negligible among his naïve (CD27-negative) cells (∼1%) (Figure 1C, right), but frequent in the CD27-positive cells (42%), which are B cells that have undergone differentiation and expansion in the periphery [23], [24].

### WASP Phenotype in Cell Types under Selection

**Figure 2:**
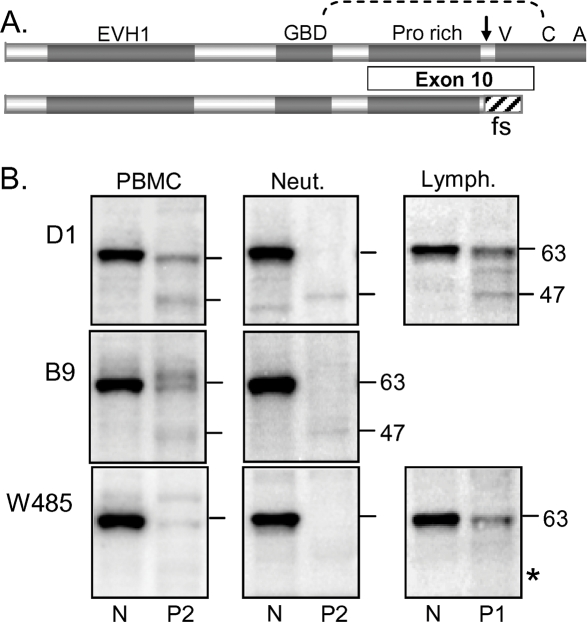
WASP in Patient T Cells Revealed by Western Blot. (A) Schematic of (top) normal WASP (502 amino acids) and (bottom) WASP encoded by the patients' germ line mutation (1305delG in exon 10, arrow) with the frameshifted region (424–443) (fs) indicated. Shown are the upstream regulatory domains: EVH-1, GBD (GTPase binding), and proline-rich region (binding sites for adapter proteins and tyrosine kinases), and the downstream verprolin homology (V) (exon 10), cofilin homology (C) (exon 11) and acidic (A) (exon 12) domains that function in generating actin filaments. The intramolecular bond of autoinhibited WASP is indicated by a dotted line. (B) PBMC, neutrophils and lymphocytes (5×10^5^ cells) of normal individuals (N) and patients 1 (P1) and 2 (P2) were stained as indicated with B9 and D1 antibodies to WASP residues 1–250 and W485 antibody to the C-terminal peptide. The numbers on the right indicate apparent mass (kDa) of WASP bands relative to marker proteins. The asterisk (*) in the W485 blot indicates the position of a 40 kDa marker protein, which was used to verify inclusion of the 47 kDa region. The blots were restained with actin antibodies, which verified equal protein loading (not shown).

The patients' germ line mutation is a single nucleotide deletion in exon 10 (1305delG) [25]. The expected effect is frameshift and premature stop within exon 10 to generate truncated protein lacking the VCA domains required for actin remodeling (Figure 2A). To study the amount and size of the WASP protein in patient cell types for which flow cytometry data indicated selection on WASP function, we studied isolated PBMC (lymphocytes plus monocytes), neutrophils and lymphocytes by Western blot. Unlike flow cytometry, which compiles data for individual single cells, Western blot reports on protein isolated from cell populations. The blots were stained with antibodies directed against different epitopes of WASP. These are antibodies B9 and D1, directed against epitopes within the N-terminal 250 amino acids, and W485, an antibody directed against residues 485-502. All three antibodies detected full length WASP bands (apparent 63 kDa) in all analyzed blood cells of normal individuals as anticipated and detected similar size WASP bands at lower levels in patient PBMC and in lymphocytes but not in patient neutrophils [26] verified in Figure 2B). In addition, D1 and B9 antibodies detected a weaker band in patient PBMC, lymphocytes and neutrophils at the approximate position expected for truncated WASP (apparent 47 kDa). This 47 kDa band was not found in cells of normal PBMC, lymphocytes or neutrophils. Blots of patient cells stained with the W485 antibody that reacts with an epitope at the extreme C-terminus failed to detect the 47 kDa protein, further indicating that it is truncated WASP. Importantly, because the patients' full size protein (∼63 kDa) was detected by the W485 antibody, the sequence at the C-terminus must be encoded by an allele with an in-frame C-terminal region in contrast with the inherited allele.

### Genetic Basis of Restored Phenotype in Patient T Cells

When mRNA of patient 2 PBMC was reverse transcribed and amplified, the product was a single band of apparent normal size and amount (not shown). Both the bulk product and randomly selected clones had the germ line frameshift-stop sequence. However, one clone that was non-randomly selected for study because of its slightly smaller insert coded for WASP with an 18 bp deletion relative to the normal sequence (17 bp deletion flanking the germ line nucleotide deletion) (detailed below). This sequence would be consistent with a somatic second site mutation. On sequencing another “small insert”, we were surprised to find a different mutation, a 20 bp deletion flanking the germ line mutation. Although a few instances of multiple somatic mutations have been described for other primary immune deficiencies no WAS patient described at that time had more than one somatic allele (Discussion).

To sequence the somatic mutations, we amplified and cloned DNA of patient 2 PBMC and T cell DNA of both brothers. A total of 14 somatic *WASP* alleles were sequenced and are shown in Table 2. It is important to note that our cloning effort was not exhaustive and did not come close to a generating a saturated sample of the population of alleles coexisting in each brother (presented later), and so Table 2 should be viewed as a subset of the brothers' alleles, which may be biased toward alleles differing from the germ line allele in length.

**Table 2:** Somatic *WASP* Alleles.

Allele#	Mutation^a^	Predicted Effect	Δ Gene length^b^	Patient	Source^c^
1	1303–1314del	424–427del	−11 bp	patient 2	T cell^G^
2	1297–1311del	422–426del	−14 bp	patient 1	T cell^G^
3	1295–1312del	421–426del	−17 bp	patient 1	T cell^G^
				patient 2	T cell^G^, PBMC^R^ (2×)
4	1301–1321del	423–429del	−20 bp	patient 2	T cell^G^
5	1299–1319del	422–428del, G429R	−20 bp	patient 2	PBMC^R^
6	1283–1315del	417–427	−32 bp	patient 1	T cell^G^ (2×)
7	1275–1328del	415–432del; P414R	−53 bp	patient 2	T cell^G^, PBMC^G^
8	1323–24insG	424–429 fs	+1 bp	patient 1	T cell^G^
9	1281–82delCT	417–424fs	−2 bp	patient 1	T cell^G^
10	1257–58insA	409–424fs	+1 bp	patient 1	T cell^G^
11	1243–44ins10bp	404–424fs	+10 bp	patient 1	T cell^G^
12	1218–1261del	396–409del; 410–424fs	−44 bp	patient 1	T cell^G^
13	1211–1239del	393–402del, 403–424fs	−29 bp	patient 1	T cell^G^
14	1113–14insC	361–424fs	+1 bp	patient 1	T cell^G^

a Nucleotides and amino acids are numbered relative to normal *WASP*. Amino acids are identified by single letter code. del, deletion; ins, insertion; fs, frameshift.

b Changes of gene and cDNA length (Δ Gene length) are expressed relative to the patients' germ line allele.

c Clones indicated by superscript^G^ were generated from genomic DNA; those indicated by superscript^R^ were generated from reverse-transcribed RNA. Except where noted (# 3, 6 and 7), each allele was cloned once.

**Figure 3:**
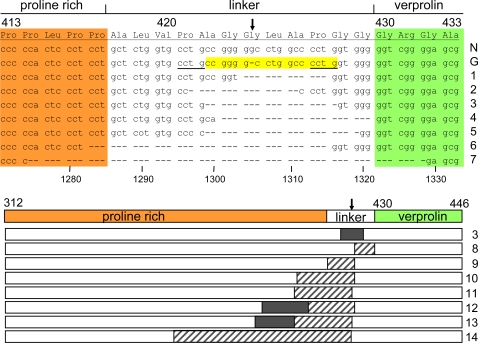
Fourteen Somatic Patient Alleles Identified by Cloning. Top: Alleles 1–7. Shown is the affected segment of exon 10 of normal *WASP* (N), the patients' germ line allele (G) and somatic alleles #1–7 (identified on the right). The arrow marks the germ line 1305G deletion. These 7 alleles encode WASP with deletions in the linker region that minimally impinge on the upstream proline-rich region (orange) and do not impinge on the downstream verprolin homology (V) domain (green). Features favorable to mutations include the high GC content and particularly nucleotides 1299–1316 (yellow), which may form a hairpin loop stabilized by five G-C bonds, and tandem 4 nucleotide repeats (underlined) that could predispose to deletion by slipped strand mispairing. Bottom: Alleles # 8–14. Shown is the entire exon 10 with 1305G marked by an arrow. Patient allele #3 is repeated to facilitate comparison. Deleted segments are shown as black, out-of-frame segments as crosshatched. Numbering is relative to normal *WASP*. Further details are in Table 2.

The detected somatic alleles are of two types. The most frequent, found in all 8 clones for patient 2 and in 4 of 11 clones for patient 1, are deletions that surround the germ line single nucleotide deletion. These encode WASP lacking 4, 5, 6, 7, 11 or 18 amino acids of the linker polypeptide that separates the proline-rich region and the verprolin homology domains (Figure 3, top). The other allele type, small insertions or deletions upstream and in one case downstream of the germ line mutation, were found in 7 of 11 clones for patient 1. These encode WASP with small out-of-frame segments and/or deletions that restore the downstream reading frame (Figure 3, bottom).

Overall, the patients' somatic alleles are highly diverse at the DNA level but have common features at the level of encoded protein. All changes are confined to the linker region, which has no known function, and limited segments of the large proline-rich region. Notably, all 14 somatic alleles restore the translational reading frame encoding the VCA domains required for WASP to function in actin remodeling in contrast to what would be expected from a random assortment of somatic mutations (Figure 3). This strongly suggests that natural selection within lymphocyte populations favors the survival and proliferation of cells expressing these VCA domain-restoring alleles over cells expressing the inherited *WASPΔVCA* allele or somatic mutations that fail to restore protein function.

### Parallel Evolution of Restored WASP in B Cell Populations *in vitro*

**Figure 4:**
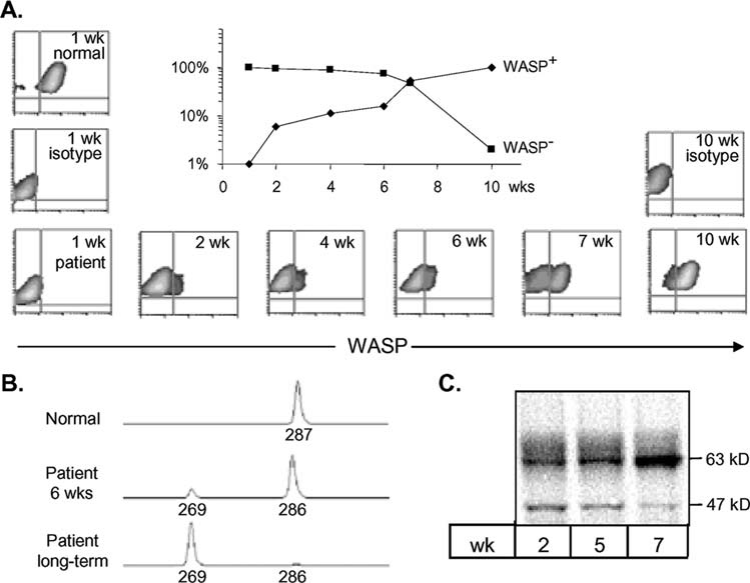
Changes in WASP Over Time of Culture of an EBV-transformed B Lymphoblastoid Cell Line of Patient 2. Cryopreserved cells from the earliest freeze of the transformed cell line were placed in culture and sampled at the indicated times. (A) WASP expression analyzed by flow cytometry after intracellular staining with B9 WASP antibody or isotype control. The insert shows the relative frequency of WASP^+^ and WASP^−^ populations on a log scale. (B) Fragment length analysis of amplified exon 10 of a normal B cell line (top), the patient B cell line at 6 weeks (middle) and after long term culture (bottom). (C) Western blot of cells harvested at 2, 5 or 7 weeks and stained with B9 antibody to reveal full-length (63 kDa) WASP and 47 kDa truncated WASP.

We also studied a B cell line from one of the brothers. This was an Epstein-Barr virus (EBV) immortalized line generated 9 years earlier from PBMC of patient 2, who was then 13 years old. When the cell line was first generated, full length WASP was found by quantitative Western blot at 15% of normal level [27], but later assays showed variably higher levels. To assess whether selection for somatic alleles also occurs *in vitro*, a remaining aliquot of the earliest cryopreserved immortalized cells was placed in culture. Flow cytometry performed 1 week later showed that 1% of cells were WASP-positive; however by 6 weeks the frequency of WASP-positive cells had increased to 16% and by 10 weeks to more than 90% (Figure 4A and insert). Nearly identical results were obtained on culturing a second aliquot of the cryopreserved cells (data not shown).

Size analysis of amplified exon 10 from the patient cell line at 6 weeks of culture showed a major fragment, 1-bp smaller than normal, consistent with the germ line allele, and a minor fragment smaller by 17-bp that represented 15% of amplified DNA (Figure 4B). The long-term cultured cells had only the latter size fragment; its sequence is identical to somatic allele #3 identified for patient T cells. Western blots showed time-dependent increase of full-length (63 kDa) WASP concurrent with time-dependent decrease of the 47 kDa apparent truncated WASP protein (Figure 4C). These findings demonstrate a change in the allele frequency within the cell line due to natural selection acting through an *in vitro* survival and/or proliferation advantage for *WASP* gene-corrected B-cells relative to germ line encoded B-cells.

### Diversity of Somatic Alleles Expressed in Patient T cells

**Figure 5:**
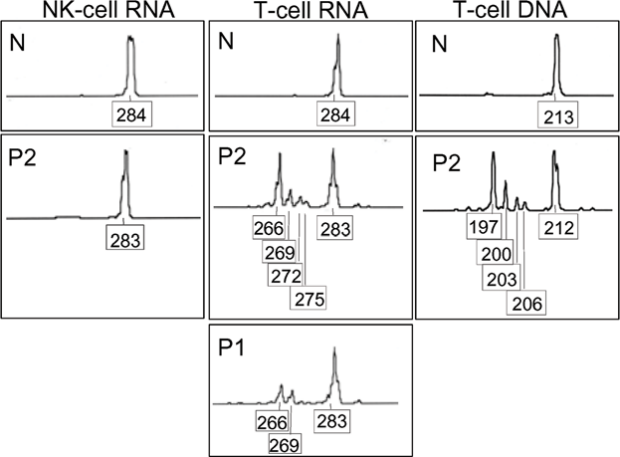
Fragment Length Analysis of Patient mRNA. Messenger RNA of T cells and NK cells was reverse-transcribed, and *WASP* was amplified from nucleotide 1151 in exon 10 to 1434 in exon 11, and the products were separated by capillary electrophoresis. Shown are products derived from RNA of NK cells (left) or T cells (middle) of a normal individual (N) or patient 2 (P2) or T cells of patient 1 (P1). Shown in the right panels are amplified exon 10 regions of genomic DNA. Fragment lengths (bp) are indicated.

The WASP protein in patient T cells could have been transcribed primarily from a single allele if most somatic mutations persisted in cells and were only occasionally transcribed. To distinguish between this possibility and that of multiple transcribed somatic alleles, we subjected amplified reverse-transcribed RNA and amplified genomic DNA to size analysis by high resolution electrophoresis. On size analysis, RNA-derived fragments from T cells and NK cells of normal healthy individuals migrated as single peaks at the predicted 284 bp (Figure 5, top row, left and middle), and patient NK-cell fragments migrated as a single 283 bp peak, consistent with the germ line allele (Figure 5, row 2, left), indicating that transcribed WASP in these cell populations comes from a single source allele. In contrast, the results for the patients clearly showed multiple peaks in T cell genomic DNA and in T cell RNA (Figure 5 rows 2, 3), demonstrating that “full length” WASP protein detected by Western blot of patient T cells is a mixture of differently “corrected” WASP proteins synthesized by cells expressing different somatic alleles.

Through high resolution electrophoresis we also aimed to describe the diversity of WASP RNA size classes. Densitometry indicated that 58% of T cell RNA of patient 2 differs in size from the germ-line allele. This is consistent with the 56% of T cells that are WASP-positive as identified by flow cytometry. Both his RNA- and DNA-derived products consisted of discrete peaks that included the germ line fragment and somatic fragments lacking 8, 11, 14 or 17 bp relative to the germ line fragment. For patient 1, individual novel peaks were less prominent, consistent with the greater diversity of this patient's somatic alleles. The “germ line” peak was broad, probably encompassing the patient's 1 bp insertions and 2 bp deletions, confounding quantitation of the percent of RNA that is somatic *vs*. germline. Conservatively, at least 2 somatic alleles made up 39% of the WASP RNA in T cells of patient 1.

Of the novel fragment size classes identified for patient 2, two, the fragments lacking 8 bp relative to the germ line allele and those lacking 14 bp, are not represented among the patient's cloned alleles. The presence of size classes of patient 2 RNA and genomic DNA that are not accounted for by the cloned alleles together with broad peaks for patient 1 indicate that the brothers have additional somatic alleles not identified in the study. These findings suggest that the identified somatic *WASP* alleles represent the “tip of the iceberg” of diversity of the patients' T cells.

## Discussion

Here we document the independent evolution of polymorphic T cell populations involving many somatic alleles in two brothers who inherited a frameshift mutation of *WASP*. As a result of selection for somatic alleles, the brothers' lymphocyte compartments are somatic mosaics and their gene-corrected cells are identifiable by WASP expression. The somatic alleles are compensatory because they (*i*) support WASP expression at approximately normal levels in certain patient cells and (*ii*) confer an apparent selective advantage in the generation of memory T cells and B cells. *WASP* mRNA in the patients' T cells had varying size, indicating that multiple somatic alleles are indeed expressed. The somatic alleles differed between the two brothers - both in the type of clones isolated and the fragment length patterns. This implies a random process at the level of generation of somatic mutations. In contrast, the quantitative enrichment of somatic alleles and their distribution among lymphocyte populations were indistinguishable between the brothers, speaking for the uniformity of the selection (enrichment) forces.

Somatic alleles were first described for primary immune deficiency for a patient with adenosine deaminase deficiency [28] and subsequently for WAS. In general, detection of a somatic mutation indicates that a new allele provides a differentiation, growth or survival advantage for the cell population(s) in which it appears. Historically, somatic alleles have been considered rare, however results obtained with newer detection methods suggest that they occur with greater frequency than previously thought, at least for genetic hematopoietic diseases (as outlined in Introduction). For example, by testing antibody stained cells by flow cytometry, an international consortium recently documented somatic *WASP* alleles, either reversions or second site mutations, in 11% of 272 WAS patients [29]. These somatic mutations of *WASP* led to a variety of enrichment patterns: T cells only were most frequent, followed by T plus NK cells or T plus B cells. The different enrichment patterns are thought to reflect the developmental stage at which the somatic allele arose (stem cell *vs.* multipotent progenitor or lineage committed progenitor) and the importance of WASP in further differentiation, proliferation and survival of the affected lineage(s). A recent publication reports on another WAS patient with multiple somatic alleles [30], and thus, the brothers described here are the second and third reported cases of multiple somatic alleles in WAS. Although their disease has features shared with single somatic allele patients, most notably the preferential enrichment of somatic alleles in memory T cells [9], [10], other important features of their phenotype are specific to multiple somatic alleles.

### Germ Line Allele Confers a Large Cost in T-Cells During Lymphopoiesis

The unique features of the brothers' disease are, first of all, the large number of somatic alleles. Cloning identified 15 alleles, 10 for one brother and 5 for the other. However, the true total is likely to be much larger as suggested by fragment analysis, which revealed size classes of alleles that are not present in the clones. A larger repertoire is also suggested by the paucity of repeat-isolations of clones with the same sequence, *i.e.*, 15 of 19 clones had unique sequence, indicating that we have not come close to a saturated sample of the population. Secondly, the somatic alleles are diverse. Among those that were cloned, six code for deletions of varying size (11–53 bp) surrounding the germ line mutation, four have small indels (+1 or −2 bp) at locations ranging from nucleotide 1113 to nucleotide 1323, and three have deletions or insertions of 10–44 bp upstream of the inherited mutation.

Whereas diversity is broad, the range of affected cells is narrow – primarily T cells and to a much smaller extent B cells. Sensitive techniques of flow cytometry, Western blot, and fragment length analysis were used to search for somatic alleles in the brothers' monocytes, neutrophils and NK cells. Only the germ line allele was found in these cells, indicating that the brothers do not have genetic instability at the *WASP* locus. Indeed, based on the collective findings, there is no reason to postulate that mutation of *WASP* in the brothers' cells occurs at anything other than the normal random rate. Instead, the much higher rate at which new somatic alleles have become detectable in these patients indicates a unique combination of timing and magnitude of selection against their inherited allele. A further unusual feature of the brothers' disease is that their blood cells that carry the germ line allele express truncated WASP. We propose that the multiplicity and diversity of their somatic alleles relative to other WAS germ line alleles is due to a deleterious effect of the presence of truncated WASP over and above the cost of absence of full-length WASP.

### Role of WASP Domains in T Cell Lymphopoiesis

Normal hematopoietic precursor cells generated in the bone marrow migrate to the thymus where they commit to the T-lineage and undergo programmed differentiation becoming first CD4/CD8 double negative (DN) thymocytes, then CD4/CD8 double positive (DP) and finally either CD4 single positive (SP) or CD8 SP thymocytes [31]. These stages differ in transcriptional, translational, migratory and proliferative activities. In mice, DN thymocytes are further distinguished as stages 1 (DN1) through 4 (DN4). Progression from DN3 to DN4 and DP depends on rearrangement of the germ line T cell receptor β-chain (TCR-β) and expression on the cell surface of pre-TCR (TCR-β associated with germ line pre-α chain). The TCR α-chain is rearranged in DP cells and successful rearrangement results in expression of a unique TCR on DP cells. The specificity of the unique TCR is the basis for positive selection and those clones that recognize self peptides in the context of self histocompatibility proteins with moderate affinity will be selected. The majority of DP cells fail to recognize self peptides and die by apoptosis. Positively selected DP cells further mature into SP cells. Both DP and SP cells undergo negative selection to deplete clones with high affinity for self antigen (*e.g.* autoreactive T cell clones); this step is the basis of central tolerance. Only the small fraction of thymocytes that survive positive and negative selection emerge from the thymus to join the pool of peripheral antigen-inexperienced (naïve) CD4 cells or CD8 cells. In ideal situations, this pool provides an adequate number of naïve T-cells with the broad diversity of TCRs required for immune protection.

### Selection against the Presence of *WASPΔVCA*

An allele similar to the patients' germ line mutation was previously studied in mice and found to be extremely deleterious in T cells. Whereas WAS patients and mice that lack WASP have modest deficits of T cells [18], [32], mice engineered to express *waspΔVCA* have profoundly decreased numbers of peripheral T cells and severely decreased thymic cellularity with 12-16% of the normal number of thymocytes [33]. On analysis of thymocyte subpopulations, the defect in *wasp*ΔVCA mice was traced to a profound block at DN3 thymocytes after TCR-β rearrangement. The extremely deleterious effect of waspΔVCA implies that the truncated protein interferes with a function crucial to survival of DN3 thymocytes or their progression to DN4 and DP thymocytes.

Zhang and colleagues postulated that waspΔVCA might interfere with the function of a redundant protein such as N-WASP or WAVE-2 [33]. N-WASP, WASP and the (three) WAVE proteins are members of the WASP protein family; each contains a VCA domain and functions in actin cytoskeletal regulation [34]. We consider the WAVE protein an unlikely candidate because WASP-WAVE homology is limited to the WASP C-terminal region. In contrast, N-WASP and WASP share homologies that include the N-terminal domains retained in WASPΔVCA. These domains function in recruiting WASP/N-WASP to sites of activation and in binding ligands that convert autoinhibited WASP/N-WASP to the active form [15], [17]. We thus anticipate that truncated WASPΔVCA encoded by the patients' germ line allele will retain the ability to bind to effector ligands, potentially competing with the ability of N-WASP to perform a redundant function. A dominant block of thymocyte maturation due to competitive inhibition of N-WASP would provide a strong selective advantage to a mutation in which the VCA domain is restored. This makes *WASPΔVCA* a dominant negative mutation in that the truncated protein interacts with effectors as the wild-type protein does, but blocks its function.

Supporting evidence for selection against the presence of WASPΔVCA is provided by a recent study in which WASP and N-WASP were deleted in murine T cells. Whereas deletion of N-WASP on a wild type background did not alter T cell development, deletion of both WASP and N-WASP severely compromised T cell development, producing a phenotype similar to that of *waspΔVCA* mice, *i.e.*, marked thymic hypocellularity and reduced number of peripheral T cells with blockage at the level of DN3 thymocytes [35]. We conclude that for *WASP* mutations resulting in no protein expression at all, N-WASP partially compensates for lack of WASP in supporting the DN3 to DP transition, but that WASPΔVCA, because it expresses the N-terminal portion of WASP, competes with the ability of N-WASP to compensate for WASP function.

Thus, we propose that, in addition to the deleterious effects conferred by the absence of functional WASP, the patients' thymocytes expressing WASPΔVCA encounter a block to differentiation/ proliferation at the human counterpart of the murine DN3 stage (described above), and the block creates an unusually strong selective advantage for thymocytes bearing beneficial somatic mutations. As a result, thymocytes with correcting somatic mutations increase in frequency as they replicate and progress through the final double negative stage to become CD4*^+^*CD8*^+^* DP thymocytes. Under this hypothesis, the population released to the periphery as CD4 or CD8 naïve T cells would be smaller than normal, but already enriched for correcting alleles.

Of note, the effect of germline *WASPΔVCA* differs quantitatively between the patients and the mouse model, in that the brothers have decreased but readily detectable numbers of WASP-negative circulating T cells whereas *waspΔVCA* mice have a nearly complete block of mature T cell generation. This discrepancy may reflect a different effect of *WASPΔVCA* in mice *vs.* humans. However, it also needs to be considered that the expression level of waspΔVCA in the mouse transgenic model approximates the normal wasp expression level. In contrast, the 47 kDa apparent truncated WASPΔVCA protein is present at low levels in patient blood cells (Figures 1 and 4). Thus the lesser impact on T cell generation in the patients may be due to lower concentration of potentially blocking truncated WASP protein, *i.e.*, a quantitative rather than a qualitative difference.

### Timing of Selection against *WASPΔVCA*

The disadvantage for *WASPΔVCA* germ line thymocytes is apparently enormous since it is sufficient to drive multiple independent somatic mutations to detectable frequencies. However, the extent to which any compensatory alleles can increase in frequency is limited because thymocytes are short-lived intermediates in T cell differentiation that lack the capacity for self renewal. The multiplicity of somatic alleles in the patients' T cells and the failure of any one allele to become fixed in the population are consistent with the selective advantage occurring at a relative late stage in T cell differentiation and explain why the absolute number of gene-corrected T cells is not sufficient to fill the naïve T cell compartment. Quite apart from mechanistic questions, the paucity of the patients' gene-corrected naïve T cells is a concern for their immune protection.

### Further Selection in the Periphery

The greater frequency of somatic alleles in the brothers' memory T-cells compared to naïve T cells indicates that further selection occurs after mature SP thymocytes enter the peripheral circulation as naïve T cells. Naïve T cell population size is controlled by thymic output and access to cytokine survival factors and, to a limited extent, by cell replication (reviewed in [36]). Thus the selection against presence of WASPΔVCA directly affects this cell population. Because memory T cells are generated from naïve T cells by antigen-induced differentiation and clonal expansion, differential clonal expansion, *e.g.*, by selection for cells with functional WASP, must explain the greater frequency of gene corrected memory T cells. Such enrichment by differential clonal expansion was previously identified for an atypical patient, a female with the genotype of a WAS carrier, but with circulating white blood cells that were a mixture of WASP-negative and WASP-positive cells due to failure of early hematopoietic selection. In contrast, memory T cells of that patient were primarily WASP-positive [37]. This second selective event occurring in peripheral T cells has been described also for WAS patients with single somatic alleles (*e.g.*, [9]), and is consistent with the known role of WASP in antigen receptor mediated (TCR-mediated) activation of naïve T cells [16], [38], [39].

The flip side of the phenomenon that naïve T cells bearing a somatic allele are preferentially recruited to become memory T cells is the selective depletion of gene-corrected naïve T cells. This explains why we don't see somatic allele enrichment in the standing naïve T cell population as one might have anticipated based on the hypothesized processes occurring in the thymus. From the perspective of immune repertoire, the patients may simply have two few gene-corrected naïve T cells for normal immune protection.

### Somatic Alleles in B Cells

B cells were also examined for the presence of somatic alleles, although the scope of the study was limited by the paucity of B cells in blood of these patients. Gene-correction was found in B cells of both brothers at levels sufficient for detection of the protein by flow cytometry. Moreover, the somatic allele(s) was strongly enriched in patient memory B cells, 40% of which were WASP-positive compared to 1% of naïve B cells. It is unclear whether the patients' precursor B cells underwent repeated selection events to generate more than one somatic allele.

Similarly to a previous report of somatic allele enrichment in a patient cell line [8], we found that *in vitro* culture of the patient B cell line reproducibly resulted in an increase of a somatic allele from 1% to close to fixation and concurrent depletion of the germ line allele and the truncated WASP protein. Whether the selected allele that we identified for the patient B cell line arose *in vitro* or *in vivo* cannot be distinguished. Regardless, these *in vitro* evolutions demonstrate a survival and/or proliferative advantage of the somatic allele over the germ line allele in competition in cell culture.

### Significance of Multiple Somatic Alleles

A principle difference exists between single *vs.* multiple somatic alleles. This difference follows directly from population genetic principles but is not obvious without this evolutionary perspective. A single somatic corrective allele can be thought of as a “jackpot” effect – a chance mutation occurring sufficiently early in maturation of a cell lineage(s) in which the germ line allele has a cost. Each patient with a “jackpot” correction will have a single somatic allele, but a different correcting allele might be found in another patient. In contrast, the presence of multiple somatic alleles in an individual patient indicates that a more deterministic process is occurring. We therefore compare the brothers with other well-characterized primary immunodeficient patients for whom more than one somatic allele was reported.

The first reported case involved the RAG1 gene (Recombination Activating Gene-1) in a patient with Omenn's syndrome, a primary immunodeficiency, and the second the CD3ζ gene in a patient with severe combined immunodeficiency (SCID) [40], [41]. Each of these patient's somatic alleles apparently benefited from a proliferative advantage relative to the defective germ line allele at a specific stage of thymocyte development. The gene-corrected cells lacked the capacity of hematopoietic stem/progenitor cells for self renewal and unlimited expansion and only partially filled the T cell compartment. We conclude that the presence of multiple somatic alleles in a primary immunodeficiency patient indicates that (*i*) a developmental stage is severely affected by the germ line allele of that patient and (*ii*) the affected developmental stage occurs post self-renewal capacity. Because population size post self-renewal will be large, many mutations will be present and strong selection will produce many different compensatory alleles. Based on the underlying population genetics, one anticipates that this will happen in all individuals who carry a particular germ line allele. This explains the similar frequency but different spectrum of somatic alleles in the two brothers. A recent report describes another WAS patient for whom 34 somatic alleles were identified [30]. This patient's germ line allele, also an exon 10 mutation, is predicted to result in a truncated WASP protein missing not only the VCA domain but also most of the proline-rich region. Once the range of cells expressing the patient's somatic alleles has been identified and the phenotype of his germ line allele clarified at the level of the protein and cell, we may well find that his case is another example of selection against the presence of truncated WASP. Interestingly, for two cases, the CD3ζ patient and the brothers with WAS, truncated protein was found in patient cells with the germ line mutation. This suggests that (*iii*) multiple somatic alleles may indicate that the germ line allele has a dominant negative effect.

Thus, a finding of multiple somatic alleles in a single individual serves to “flag” an inherited allele harboring unique information. It has potential to contribute toward identifying tightly regulated maturational steps and providing clues to the specific function of the gene product at the particular developmental stage.

## Methods

### Patients

Patient 1, a white male, was 25 years old at the time most of these studies were done. The diagnosis of the WAS was made in infancy because of petechial hemorrhages and a positive family history. At 3 years of age a splenectomy was performed because of persistent thrombocytopenia. His subsequent growth and development were normal. He developed mild eczema of the extremities at an early age. During his teenage years he had recurrent *Molluscum contagiosum* infection of the eczematous areas of his skin. At age 16 he developed moderate to severe symmetrical arthralgias with joint swelling and immune complex uveitis. At age 21 he complained of low back pain. A diagnosis of sacral osteomyelitis was established, due to *Staphylococcus aureus*, and successfully treated with antibiotics.

The brother, patient 2, was 22 years old at the time most of these studies were done. Appearance of petechiae in the neonatal period led to the diagnosis of the WAS. A splenectomy was performed at age 3 years. He continued thereafter, like his brother, to take daily antibiotic prophylaxis and intravenous gamma globulin at 4-week intervals. Except for otitis media, he did not have serious infections. He was admitted to the hospital at age 7 because of severe otitis externa, which was treated with carbenicillin and gentamycin. He never had significant eczema. Subsequent to the experimentation reported here, patient 2 developed non-Hodgkin T cell lymphoma. He was successfully treated by cord blood transplantation.

### Cell Analysis and Isolation

Blood samples were collected with informed consent from the patients and healthy control individuals using acid-citrate-dextrose (NIH formula A) as anticoagulant under protocols approved by the Institutional Review Board of the Immune Disease Institute. The patients in this manuscript have given written informed consent (as outlined in the PLoS consent form) to publication of their case details. The blood was examined immediately or after overnight shipment at ambient temperature. Cell populations were quantified using the Max-M cell counter (Coulter Corp, Hialeah, FL) and by flow cytometry after staining aliquots of whole blood with combinations of marker antibodies as described [18]. To isolate specific cell populations, the blood was processed by differential centrifugation as described [26]. Density centrifugation on Histopaque 1077 (Sigma, St Louis, MO) was used to separate the peripheral blood mononuclear cells (PBMC) (lymphocytes plus monocytes) from the neutrophils. Lymphocytes were isolated from PBMC by depleting monocytes by adherence to serum-coated plates. Lymphocytes were isolated from PBMC by negative immunomagnetic selection and natural killer lymphocytes (NK lymphocytes) were isolated by positive immunomagnetic selection with CD56 antibody (reagents from Dynal Biotech, Oslo, Norway).

### Flow Cytometry

PBMC (5×10^5^) in 50 µl phosphate buffered saline with 1% fetal calf serum (PBS-FCS) or whole blood (100 µl) were surface-stained with phycoerythrin (PE) or phycocyanin-5 (PC5)-labeled CD3, CD19, CD4, CD8, CD16, CD27, CD45RA and CD56, or IgG1 control antibodies (Beckman Coulter, Hialeah, FL) for 15 min at ∼22°C. Whole blood was lysed with FACS™ Lysing Solution (BD Biosciences, Palo Alto, CA) for 15 min at ∼22°C. After washing with 3 ml PBS-FCS, the cells were incubated in 250 µl of Fix/Perm solution (Pharmingen, San Diego, CA) for 20 min on ice, washed twice, stained with 5 µg/ml of B9 anti-WASP monoclonal antibody (Santa Cruz, Santa Cruz, CA) or IgG2a isotype control (Pharmingen) for 30 min on ice, and washed twice with Perm/Wash solution. The cells were incubated with 10 µg/ml of fluorescein isothiocyanate- (FITC-) conjugated goat antibody to mouse IgG2a (Southern Biotechnology, Birmingham, AL) for 30 min, washed twice with Perm/Wash solution, re-suspended in 50 µl PBS and analyzed immediately on the FACS Calibur by collecting 10,000 cells/sample. Individual cell populations were separately evaluated by gating based on forward and side scatter and/or expression of surface markers.

### Western Blots

Isolated PBMC, lymphocytes and neutrophils were washed, treated with protease inhibitors and lysed with an SDS solution containing 2-mercaptoethanol as described [26]. The lysates were fractionated by SDS electrophoresis, transferred electrophoretically to polyvinylidene difluoride (PVDF) membrane, which was blocked, washed and incubated with affinity-purified rabbit W485 antibody to WASP [27] or mouse D1 or B9 monoclonal antibodies to WASP (Santa Cruz Biotechnology, Santa Cruz, CA). After further washes, the membrane was incubated with ^125^I labeled goat antibodies to rabbit immunoglobulin or rabbit antibodies to mouse immunoglobulin [26]. Where indicated, WASP bands were quantified using the Storm 860 Imager and Image Quant (Molecular Dynamics, Sunnyvale, CA).

### DNA and RNA Analysis

Genomic DNA was isolated using QiaAmp DNA purification kit (Qiagen, Valencia, CA). *WASP* exon 10 was amplified for cloning with intron primers [42], and PCR products were gel-purified and cloned into pSTBlue-1 (Novagen, Darmstadt, Germany). RNA was isolated using Trizol (Invitrogen, Carlsbad, CA) and was reverse-transcribed (2 µg in 20 µl) with oligo-dT (1 µg) Superscript II (200 units) (Promega, Madison, WI) at 45°C for 50 min. *WASP* cDNA was amplified for cloning from *WASP* 906 to 1706 with 5′-acgacttcattgaggaccag-3′ and 5-tgagtgtgaggaccaggcag-3′ (1 µM of each), 2 mM MgSO_4_, 0.2 mM dNTPs, and 2.5 U Platinum Taq High Fidelity polymerase (Invitrogen) at 95°C for 2 min, 35 cycles at 95°C for 30 s, 57°C for 30 s, 72°C for 1 min with 10 min extension at 72°C. For high resolution fragment length analysis, RNA was reverse-transcribed, and cDNA and also genomic DNA were amplified using 6-carboxyfluorescein (FAM) labeled 5′-tccagctactggacgttctg-3′ together with unlabeled 5′-accagtccctctgagctctg-3′ (cDNA) or 5′-cttgttcagctgaattccctgcag-3′ (T-cell genomic DNA) or 5′-tcctgacttagacgggacac-3′ (B-cell genomic DNA). The products were separated by capillary electrophoresis
